# Breast Cancer Epidemiology and Survival Analysis of Shenyang in Northeast China: A Population-Based Study from 2008 to 2017

**DOI:** 10.1155/2022/6168832

**Published:** 2022-10-18

**Authors:** Hongbo Su, Xun Li, Yi Lv, Xueshan Qiu

**Affiliations:** ^1^Department of Pathology, The First Hospital of China Medical University, Shenyang 110001, China; ^2^Section for Chronic Disease Control, Shenyang Center for Disease Control and Prevention, Shenyang 110001, China

## Abstract

**Background:**

To investigate the status of breast cancer incidence, trends, and survival in women in urban Shenyang from 2008–2017 using large Cancer Registry data.

**Methods:**

Breast cancer incidence and mortality data were abstracted from the Shenyang Cancer Registry between 2008 and 2017. The crude and age-standardized incidence and mortality rates were calculated for each year. Average annual percentage changes (AAPC) were used to describe the change over time.

**Results:**

A total of 14,255 out of 18,782,956 women were diagnosed with breast cancer between 2008 and 2017 in urban Shenyang. The overall crude and age-standardized incidences were 75.89 and 43.42 per 100,000, respectively. The crude incidence increased from 61.93 per 100,000 in 2008 to 90.07 per 100,000 in 2017, with an AAPC of 5.10%. The crude mortality increased from 11.41 per 100,000 in 2008 to 17.29 per 100,000 in 2017, with an AAPC of 4.60. The highest age-specific incidence occurs in the 55–59 year age group at a rate of 140.67 per 100,000. During the study period, 2,710 women died from breast cancer. The overall crude and age-standardized mortality rates were 14.43 and 7.43 per 100,000, respectively. The highest age-specific mortality occurs at 80–84 years old at a rate of 57.57 per 100,000. The 3-year and 5-year survival rates for female breast cancer in urban Shenyang from 2008 to 2013 were 85.61% and 77.39%, respectively, and both declined with age.

**Conclusion:**

The incidence and mortality rates of breast cancer in Shenyang increased over time. Screening and control strategies should be enhanced, especially for perimenopausal females.

## 1. Background

Breast cancer is one of the most common malignancies in women worldwide, with approximately 2.1 million newly diagnosed cases in 2018 accounting for 11.6% of all new cancer cases. Breast cancer is associated with 0.63 million deaths worldwide, accounting for 6.6% of all cancer-related deaths in women [1]. According to GLOBOCAN 2018, breast cancer is the most frequently diagnosed cancer in the vast majority of countries (154 of 185) and is also the leading cause of cancer death in over 100 countries [1]. The American Cancer Society 2020 reported a slight increase in breast cancer incidence rates by 0.3% per year and a decrease in mortality rates from 2013–2017 by 1.3% per year [2].

China, the world's most populous country, has experienced an increase in the reported incidence and prevalence of breast cancer [2], despite improvements in health care and increasing funding for cancer control. Currently, breast cancer is the leading cancer in Chinese women, with 367,900 new cases accounting for 8.6% of all new cancer cases (including men and women) and 19.2% of new cancer cases in women and is associated with 97972 deaths according to the GLOBCAN 2018 [3]. The breast cancer mortality rate has been observed to increase during 1990–2015, which is partly attributed to an increasingly aging population [1,4]. The age-standardized incidence and mortality rates in China are reported to be 36.1 per 100,000 and 8.8 per 100,000, respectively, according to The Global Cancer Observatory 2020. The age-standardized incidence rate per 100,000 people by the Chinese standard population was 1.4 times as high in urban areas as in rural areas (35.75 vs. 25.53 cases per 100,000 people) [1].

Breast cancer incidence and mortality are strongly associated with age. For female breast cancer, the peak age for incidence can vary in different regions; for example, 45–50 years old for China, Japan, and South Korea, and around 60 years old for Europe and the USA [5]. Women younger than 50 years usually have a lower breast cancer incidence. The older age group has a higher age-specific mortality rate [6,7].

Shenyang is a major city in Northeast China, whose population increased from 5.27 million in 2005 to 7.22 million in 2020. The epidemiology of breast cancer in Shenyang may, to some extent represent the epidemiology of the Northeastern area of China and provide some clues on the overall prevalence in Northeast China. In addition, Shenyang is a developing Asian city, which shares many similarities with other developing Asian cities [8]. Therefore, the epidemiology and disease control of breast cancer in Shenyang can provide information and potential guidance for similar Asian cities. The changes in breast cancer screening and treatment strategies in modern times could greatly impact breast cancer incidence and trends. The global data above may not accurately represent the breast cancer status in the major cities in our country. To better understand the status and trends of breast cancer in our country, we investigated the status and trends in female breast cancer epidemiology from 2008 to 2017 in urban Shenyang.

## 2. Methods

### 2.1. Incident Cases

Cancer incidence data from 2008 to 2017 was provided by the Shenyang Cancer Registry, which is one of the population-based cancer registries in China and was established officially by the Shenyang Center for Disease Control and Prevention in 1998. The cancer data of the Shenyang Cancer Registry is recognized by the International Association of Cancer Registries. Breast cancer incident cases registered from 2008 to 2017 were identified according to the International Classification of Diseases for Oncology (third edition) using topography code C50.

### 2.2. Cases of Death

Cancer death data from 2008–2017 was provided by the Shenyang Municipal Bureau of Public Security. Breast cancer deaths registered from 2008 to 2017 were identified based on the 10th version of the International Classification of Diseases.

### 2.3. Statistical Analysis

#### 2.3.1. Crude Incidence and Mortality

Crude incidence per 100,000 was calculated by dividing the total number of new cases of female breast cancer reported each year by the average female population in that year multiplied by 100,000. Crude mortality per 100,000 was calculated by dividing the total number of women dying from breast cancer each year by the average female population in that year multiplied by 100,000. Incidence and mortality rates were calculated for each year throughout the 10-year study period. The overall incidence and mortality were also calculated for the whole study period. The overall percentage change and average annual percentage change (AAPC) were calculated with joinpoint regression modeling using Joinpoint Trend Analysis Software [9].

This method identifies joinpoints that connect distinct line segments, thus allowing for a succinct characterization of changes in data over time. A maximum of 3 joinpoints were allowed based on the number of data points. We calculated the annual percentage change for each segment, and the AAPC across the entire trend line represents the weighted average of the APC from each constituent segment, where the weights are proportional to the length of the segment over the follow-up period.

#### 2.3.2. Age-Standardized Incidence and Mortality

The incidence and mortality in each year throughout the 10-year study period as well as the overall incidence and mortality for the whole study period were standardized by the standard world population (WHO, 2000) [10,11]. The overall percentage change and AAPC were calculated using the standard formula as mentioned earlier.

#### 2.3.3. Age-Specific Incidence and Mortality

The study population was divided into 18 age groups from 0 to >85 years with 5-year intervals. The age-specific incidence and mortality rates for each group were calculated using the standard formulae. The overall percentage change and the AAPC were calculated using the standard formula as mentioned earlier.

#### 2.3.4. 3-Year and 5-Year Survival Rates

The survival patients who were diagnosed with breast cancer during the period of 2008–2016 were contacted by telephone and followed up once a year. By the end of 2019, a total of 12377 patients had been followed up for 3 years, and a total of 8868 patients had been followed up for 5 years. The new dead cases of breast cancer each year were added to the tumor incidence database, and the survival time of each case was calculated by the date of death minus the date of diagnosis. The survival rates at 3 and 5 years were calculated by the Kaplan–Meier estimators. Censoring was considered lost to follow-up and the end of the study period.

#### 2.3.5. Statistical Analysis Software

SPSS version 22.0 (IBM Corp., Armonk, N.Y., USA) was used for statistical analysis. Joinpoint Trend Analysis Software was used to calculate AAPC [9]. *p*-values < 0.05 were considered statistically significant.

## 3. Results

### 3.1. Breast Cancer Incidence

#### 3.1.1. Incidence of Breast Cancer in Urban Shenyang from 2008 to 2017

**Figure 1:**
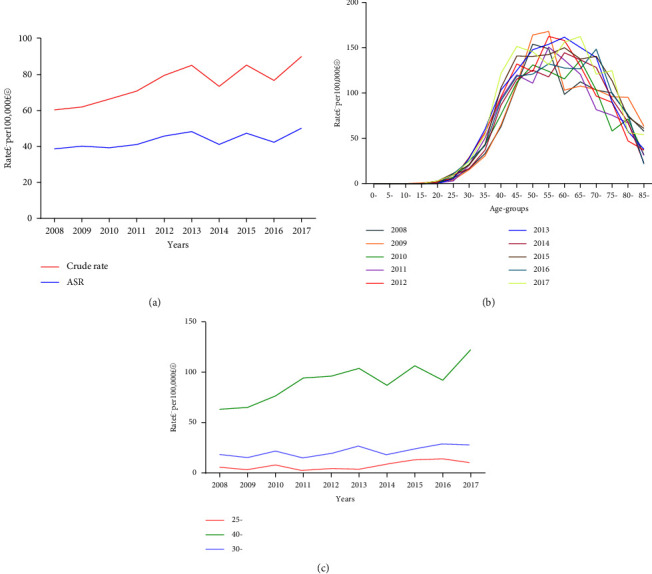
Incidence of female breast cancer in urban Shenyang in 2008–2017. (a) Overall incidence. (b) Age-specific incidence rates. (c) Age-specific incidence trends of female breast cancer for three age groups over time. The 25–29 years, 40–44 years, and the 30–34 years age groups showed the most increase in incidence over time. ASR, age-standardized incidence rate.

The total number of women in urban Shenyang between 2008 and 2017 was 18,782,956. There were 14,255 newly diagnosed breast cancer cases during this period. Correspondingly, the overall crude incidence was 75.89 per 100,000, and the overall age-standardized incidence was 43.42 per 100,000. The crude incidence increased by 53.17% over the study period, with an AAPC of 5.10% (95% CL: 2.2–8.2%, *p* = 0.001) and a joint point at the year 2013. The increasing trend of 2007–2013 was smaller than that of 2013–2017, with the APC of 2.7% (95% CL: −1.8–7.4%, *p* = 0.182) and 8.2% (95% CL: 1.6–15.2%, *p* = 0.024), respectively. The age-standardized incidence increased by 32.62% over the study period, with an AAPC of 3.50% (95% CL: 0.1–7.0%, *p* = 0.046) and a joint point at the year 2013. The APC of 2007–2013 was -0.3% (95% CL: −5.4–5.1%, *p* = 0.900) without showing a significant increasing trend, whereas the APC of 2013–2017 was 8.3% (95% CL: 0.6–16.7%, *p* = 0.039), showing an obvious increasing trend. Both the crude incidence and the age-standardized incidence showed an increasing trend (Tables 1–3, Figure 1(a)).

#### 3.1.2. Age-Specific Incidence of Breast Cancer in Urban Shenyang from 2008 to 2017

The age-specific incidence of female breast cancer was relatively low before the age of 25 years and increased significantly afterward. The incidence peaked in the 55–59 years age group, with an incidence of 140.67 per 100,000. The 60–64 years age group had the second highest incidence, followed by the 65–70 years age group, with the incidence of 137.38 per 100,000 and 137.02 per 100,000, respectively. After the age of 65 years, the incidence gradually declined. The age groups of 25–29 years, 40–44 years, and 30–34 years showed an increase in the trend of incidence over time, and the age-specific incidence of these groups increased over the ten-year period with an AAPC of 13.6% (95% CL: 0.5–28.4, *p* = 0.043), 7.1% (95%CL: 3.0–11.3, *p* = 0.001), 6.1% (95% CL: 1.6–10.8, *p* = 0.013), respectively (Table 4, Figure 1(b) and 1(c)).

### 3.2. Breast Cancer Mortality

#### 3.2.1. Mortality of Breast Cancer in Urban Shenyang from 2008 to 2017

**Figure 2:**
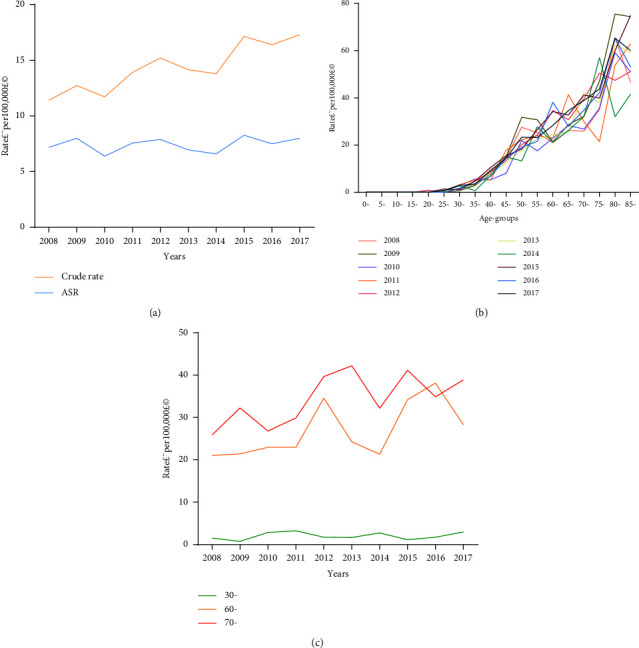
Mortality of female breast cancer in urban Shenyang in 2008–2017. (a) Overall mortality rates; (b) age-specific mortality rates; (c) age-specific mortality trends in three age groups over time. The 30–34 years, 70–74 years, and 60–64 years age groups showed the most increase in mortality over time. Abbreviations: ASR, age-standardized mortality rate.

A total number of 2,710 women died from breast cancer during the period 2008–2017. The overall crude mortality was 14.43 per 100,000, and the overall age-standardized mortality was 7.43 per 100,000. The crude mortality increased by 51.53% over this period, with an AAPC of 4.6% (95% CL: 0.8–8.5, *p* = 0.016) with a joint point in the year 2012. The increasing trend of 2012–2017 was slightly smaller than that of 2008–2012, with the APC of 3.8% (95% CL: −1.9–9.9%, *p* = 0.150) and 5.5% (95% CL: −2.7–14.3%, *p* = 0.150), respectively. Meanwhile, the age-standardized mortality increased by 11.44% over this period, and the AAPC was 0.8% (95% CL: −1.5–3.1, *p* = 0.457) (Tables 5–7, Figure 2(a)).

#### 3.2.2. Age-Specific Mortality of Breast Cancer in Urban Shenyang from 2008 to 2017

The age-specific mortality rate of female breast cancer was relatively low before 35 years of age. For women aged 0–19 years, the mortality rate was 0 per 100,000. After 35 years of age, mortality increased dramatically. The mortality rate peaked in the 80–84 years age group, with a mortality rate of 57.57 per 100,000. The >85 year age-group ranked second, and the 75–79 years age-group ranked third, with a mortality rate of 57.54 per 100,000 and 41.18 per 100,000, respectively. The age groups of 60–64 year, 30–34 years, and 70–74 years demonstrated increasing trends of mortality over time; the age-specific mortality of these groups increased over the ten-year period with AAPC of 5.0% (95% CL: 0.3–10.0, *p* = 0.040), 4.1% (95% CL: −7.9–17.8, *p* = 0.468), and 4.1% (95%CL: 0.6–7.7, *p* = 0.027), respectively (Table 8, Figures 2(b) and Figure 2(c)).

### 3.3. Breast Cancer Survival

#### 3.3.1. Three-Year Survival Rates for Breast Cancer in Urban Shenyang from 2008 to 2016

A total number of 12377 women were diagnosed with breast cancer during the period of 2008–2017 and were followed up for 3 years or more. There were 10596 cases with survival periods longer than 3 years, with a 3-year survival rate of 85.61%.

**Figure 3:**
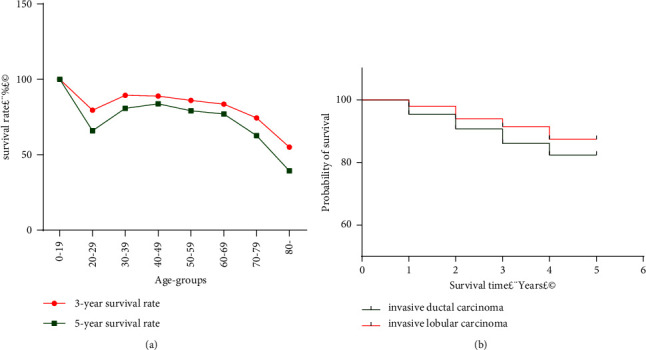
(a)The 3-year and 5-year survival rates for female breast cancer in urban Shenyang in 2008–2017. (b)The 3-year and 5-year survival rates of two common breast cancer histological types in urban Shenyang from 2008 to 2013 (the log-rank test *p* = 0.0630).

For women aged 0–19 years, the 3-year survival rate was 100%; subsequently, it decreased to 86.67% in the 20–29 years age group, which was relatively low among all age groups. The 3-year survival rate of the 30–39 years age group increased to 89.93%, which was the highest among all age groups, then it gradually decreased with age. After the age of 69 years, the 3-year survival rate showed a rapid downtrend. The lowest 3-year survival rate observed in the age group above 80 years was 53.68% (Table 9, Figure 3(a)).

#### 3.3.2. Five-Year Survival Rates for Breast Cancer in Urban Shenyang from 2008 to 2014

Among all the follow-up cases, 6863 out of 8868 breast cancer patients survived for more than 5 years, with a 5-year survival rate of 77.39%.

With increasing age, the 5-year survival rates displayed a similar pattern as the 3-year survival rates. After the age of 19, the 5-year survival rate decreased to a relatively low level of 68.18%, as observed in the 20–29 years age group. Thereafter, the rate increased to the highest level of 83.24% in the age group of 40–49 years. The 5-year survival rate declined with age, gradually after 49 years of age and dramatically after the age of 69 years. The lowest rate was observed in the age group above 80 years, which was 42.02% (Table 9, Figure 3(a)).

#### 3.3.3. The 3-Year and 5-Year Survival Rates of Two Common Breast Cancer Histological Types in Urban Shenyang from 2008 to 2014

Invasive ductal carcinoma and invasive lobular carcinoma are the two common histological types of breast cancer. Pathological data of 4487 invasive ductal carcinomas and invasive lobular carcinomas were obtained for all cases followed up for 5 years, which included 4288 invasive ductal carcinomas and 199 invasive lobular carcinomas. Among the patients with invasive ductal carcinoma, 3695 patients survived at least 3 years, and 3405 patients survived at least 5 years. Correspondingly, the 3-year and 5-year survival rates were 86.17% (95% CL: 85.14–87.20) and 79.41% (95% CL: 78.20–80.62), respectively. The Kaplan–Meier and Cox multivariable regression analyses were performed to compare the difference between the survival rates of two histological types. Among the 199 cases with invasive lobular carcinoma, 182 cases survived more than 3 years, and 170 cases survived more than 5 years. Correspondingly, the 3-year and 5-year survival rates were 91.46% (95% CL: 87.58–95.34) and 85.43% (95% CL: 80.53–90.33), respectively. After adjusting for age effect, the survival difference between invasive ductal carcinoma and invasive lobular carcinoma was statistically significant (the log-rank test, *p* = 0.039, *χ*^2^ = 4.275, Cox regression analysis, Hazard ratio:1.46 (*p* = 0.045)) (Tables 10 and 11, Figure 3(b)).

## 4. Discussion

The epidemiologic status of female breast cancer in urban Shenyang between 2008 and 2017 was investigated utilizing data from the Shenyang Cancer Registry. Our study revealed a relatively high female breast cancer incidence and mortality, as well as an upward trend from 2008 to 2017 in Shenyang.

Both the overall crude and age-standardized incidences of breast cancer in urban Shenyang were considerably high in China. The age-standardized incidence and mortality rates for breast cancer in China are reported to be 36.1 per 100,000 and 8.8 per 100,000, according to The Global Cancer Observatory 2020. In urban Hebei province, the crude incidence of breast cancer in 2013 was 45.92 per 100,000, and the age-standardized incidence was 35.54 per 100,000 [12]. In urban Beijing, the crude and age-standardized incidence rates in the period 2008–2012 were 68.50 and 40.64 per 100,000, respectively [13]. However, when compared with the worldwide breast cancer incidence rate at the same time, Shenyang's rates are in the middle range. According to GLOBOCAN 2018, the age-standardized incidence rate of breast cancer for Eastern Asia is 39.2; for the world, it is 46.3 per 100,000; for developing and developed countries, it was 31.3 and 54.4 per 100,000, respectively [1].

The age-specific incidence rates of breast cancer in urban Shenyang rose with advancing age after 21 years old, reaching a peak at 55–59 years, and then decreasing after 65 years old. Interestingly, the peak age for breast cancer incidence in urban Shenyang occurs over time in an older age group. The highest incidence was observed in the 50–54 age group in 2008, versus the 65–69 age group in 2017. Yang's study demonstrated that, in urban Beijing, the age group with the highest incidence was 60–64 years during 2004–2008 [14]. The high incidence of breast cancer in these age groups could be related to menopause and changes in body hormone levels. A worldwide collaboration analysis of data from 117 epidemiological studies from 35 countries, including 118,964 women with breast cancer, none of whom had used postmenopausal HRT, demonstrated a consistent finding of a greater risk of breast cancer among premenopausal women when compared with postmenopausal women of the same age. In analyses of postmenopausal women in this same pooled analysis, the relative risk of breast cancer increased by a factor of 1.029 for every delayed year of menopause [15].

In China, breast cancer incidence has risen gradually since the 1980s [16]. During the period 1998–2007, the crude incidence in China's urban population (164 million) increased from 36.2 per 100,000 in 1998 to 51.2 per 100 000 in 2007, with an annual rate of 3.9%; while in the rural population (55 million), crude incidence has increased from 10.4 per 100,000 to 19.6 per 100,000 with an annual rate of 6.3% [17]. In Shenyang, both crude incidence and age-standardized incidence increased over the period of 2008–2017. This rising trend of breast cancer incidence rates can be observed in the entire country, with the age-standardized incidence increasing from 22.18 per 100,000 in 2003 to 25.89 per 100,000 in 2011 [18]. Female breast cancer incidence in Shanghai increased by 141.2% from 1973 to 2012 [19]. Although the increase in new cases was alarming, this trend can be partially attributed to the changes in reproductive patterns and the aggressive screening efforts [20].

Among all age groups in our study, the 40–44 years age group showed the fastest increase in the trend of incidence over time, which is associated with the perimenopause age in China. The incidence in the 25–29 years age group also increased dramatically, with an average increase rate of 72.44% and an AAPC of 11.1% over the ten-year period. A similar phenomenon has also emerged in other Asian regions such as Japan, Hong Kong, and Taiwan, accompanied by the westernization of lifestyle after the Second World War [21]. This phenomenon emphasizes the fact that women of newer generations face a higher risk of breast cancer than those of earlier generations.

Both the overall crude and age-standardized mortality rates of breast cancer in urban Shenyang were relatively high compared to other areas in China. The crude and age-standardized mortality rates of breast cancer in China in 2013 were 9.74 per 100,000 and 6.34 per 100,000, respectively [22]. The crude and age-standardized mortality rates of urban Hebei province in 2013 were 13.30 and 9.84 per 100,000, respectively [12]. But when compared to the world mortality rates for breast cancer in the same period, Shenyang's age-standardized rates were relatively lower. According to GLOBOCAN, in 2018, the age-standardized mortality rate of breast cancer worldwide was 13.0 per 100,000; in Eastern Asia, it is 8.6 per 100,000. The breast cancer mortality in urban Shenyang rose with age and displayed no declining trend. The mortality peak is at 80–84 years old. This pattern suggests that advancing age has a poorer prognosis for breast cancer.

The mortality rates of breast cancer in China have been steadily rising, which is expected to continue in the future. The age-standardized mortality rate in China increased from 4.40 per 100,000 in 1991 to 5.31 per 100,000 in 2011 [23]. South Asia, including China, has shown a large increase in breast cancer deaths between 1990 and 2017 [24]. In Shenyang, both the crude mortality and the age-standardized mortality rates increased during 2007–2018. The up-trend of age-standardized mortality was slower than the crude mortality rates, which indicated that the observed increasing breast cancer mortality could partly be attributed to the aging population in our country. This is also suggested by other literature [25–27].

The 3-year and 5-year survival rates for female breast cancer in urban Shenyang were 85.61% and 77.39%, respectively. The age-standardized 5-year relative survival rate pooled for all 17 registries all over China from 2003 to 2005 was 73% [19]. However, it was still lower than the rate for developed countries such as the USA, where it was 88% (for all stages and races) [5]. In general, survival rates decrease with increasing age. However, comparatively low survival rates were observed in the age group of 20–29 years for both the 3-year and 5-year survival rates. The reason for this phenomenon could be a higher malignant potential of the common pathological and molecular subtypes of breast cancer in this young age group [2,28], which deserves further study.

Breast cancer is associated with a lifetime of exposure to risk factors [29]. There has been an assumption that the lower breast cancer incidence rates in Asian populations are due to the Asian lifestyle. However, as lifestyle become more westernized, the staple foods of the traditional Chinese diet have translated from grain, rice, vegetables, soybean, pork, or fish to energy-dense foods such as fat, dairy products, and animal protein [30,31]. Urbanization has contributed to a more sedentary lifestyle, an increased obesity rate, earlier menarche, and later menopause in women. Meanwhile, the inclusion of more women in the workforce, the rising cost of living in the cities, as well as the one-child policy in China, have resulted in reproductive pattern alterations, such as declining birth rates, delayed childbearing, and decreased breastfeeding [31,32]. In addition, aging is a major risk factor in cancer's epidemiological pattern. The incidence of cancer increased rapidly with increasing age.

Based on our findings, greater effort should be made to improve primary prevention strategies for women in Northeast China, such as encouraging weight control, breast feeding, and quitting drinking as well as smoking. Our study suggested that the highest incidence of breast cancer occurred during the menopausal period, therefore more attention should be paid to menopausal women, especially those with decreased circulating estrogen levels, with the option of drug intervention if necessary. Persistent efforts to provide high-quality screening, accurate diagnosis, and advanced treatment may also be effective in controlling breast cancer incidence and mortality. In addition, the novel observation from our study is that there is an increasing incidence of breast cancer in young women in urban areas. Therefore, it is important for clinicians to pay more attention to the prevention and treatment of breast cancer in young women.

Our study has some limitations. Herein, we just described the incidence, mortality, survival, and trends of breast cancer in urban Shenyang. First, it is a retrospective registry-based study, as some subjects may be lost to follow-up if they leave the registry. Our data, however, have a large study population and good follow-up for the population. Second, as we described an uptrend in breast cancer in the region, the underlying reasons and potential risk factors for the uptrend require further investigation in the future. Thirdly, we were not able to collect important clinical data such as TNM, histogram grading, and hormone receptor status for our cohort. We will hopefully have the capacity to investigate these important factors in the future study.

## 5. Conclusion

This study has described the overall and age-specific incidence, mortality, and trends of breast cancer in urban Shenyang. In urban Shenyang, female breast cancer incidence and mortality were relatively high and showed an upward trend from 2008 to 2017. These breast cancer statistics can help guide health policy and care for breast cancer in major China cities like Shenyang.

## Figures and Tables

**Table 1:** Incidence of female breast cancer in urban Shenyang in 2008–2017.

Year	Population	Cases	Crude rate	ASR
All	18,782,956	14,255	75.89	43.42
2008	1,761,826	1,091	61.93	39.52
2009	1,774,839	1,158	65.25	42.16
2010	1,835,562	1,238	67.45	39.81
2011	1,897,625	1,233	64.98	37.29
2012	1,906,605	1,402	73.53	41.63
2013	1,901,339	1,321	69.48	38.85
2014	1,921,490	1,425	74.16	41.37
2015	1,913,126	1,747	91.32	51.08
2016	1,921,961	1,762	91.68	50.94
2017	1,948,583	1,848	94.84	52.41

Abbreviations: ASR, age-standardized mortality rate.

**Table 2:** APC and AAPC of the crude incidence of female breast cancer in urban Shenyang in 2008–2017.

Year	APC			AAPC			Join point
	APC (95%CI)	*t* value	*p* value	AAPC (95%CI)	*t* value	*p* value	
2008–2013	2.7 (−1.8, 7.4)	1.5	0.182	5.1 (2.2, 8.2)	3.4	0.001	2013
2013–2017	8.2 (1.6, 15.2)	3.2	0.024				

**Table 3:** APC and AAPC of the age-standardized incidence of female breast cancer in urban Shenyang in 2008–2017.

Year	APC			AAPC			Join point
	APC (95%CI)	*t* value	*p* value	AAPC (95%CI)	*t* value	*p* value	
2008–2013	−0.3 (−5.4, 5.1)	−0.1	0.900	3.5 (0.1, 7.0)	2.0	0.046	2013
2013–2017	8.3 (0.6, 16.7)	2.8	0.039				

**Table 4:** Age-specific incidence of female breast cancer in urban Shenyang in 2008–2017.

	2008	2009	2010	2011	2012	2013	2014	2015	2016	2017	2008–2017 (overall)	AAPC	95%CI	*P* Value	Join point
All	61.92	65.25	67.45	64.98	73.53	69.48	74.16	91.32	91.68	94.84	75.73	—	—	—	—
0–4	0	0	0	0	0	0	0	0	0	0	0	—	—	—	—
5–9	0	0	0	0	0	0	0	0	0	0	0	—	—	—	—
10–14	0	0	0	0	0	0	0	0	0	0	0	—	—	—	—
15–19	0	0.89	0	0	0	0	0	0	0	0	0.14	—	—	—	—
20–24	0	2.3	0.78	0.82	2.8	0	1.21	4.08	0	3.07	1.4	—	—	—	—
25–29	6.06	3.41	7.85	2.38	4.4	4.02	8.87	12.8	13.93	10.45	7.15	13.6	(0.5–28.4)	0.043	—
30–34	17.59	15.49	21.43	14.84	19.66	26.38	17.73	23.89	28.74	27.77	21.66	6.1	(1.6–10.8)	0.013	—
35–39	34.47	33.63	44.47	45.98	41.77	47.03	40.15	46.19	47.85	58.35	43.96	4.4	(1.7–7.2)	0.005	—
40–44	64.88	67.85	79.1	83.11	90.38	71.03	86.33	115.43	119.12	117.49	87.72	7.1	(3.0–11.3)	0.001	2013
45–49	114.46	117.75	110.63	108.49	123.75	102.81	117.04	162.98	146.03	152.58	124.22	3.7	(0.7–6.9)	0.022	—
50–54	157.97	174.78	132.03	101.05	111.57	119.98	133.09	139.05	141.36	156.47	135.22	−0.3	(−4.6–4.3)	0.901	—
55–59	153.43	174.56	125.74	137.36	149.05	121.47	121.28	145.58	153.79	146.16	140.67	−0.7	(−3.7–2.4)	0.613	—
60–64	92.8	105.94	113.75	122.9	140.96	122.32	126.53	167.48	156.5	159.39	137.02	5.9	(3.7–8.2)	<0.001	—
65–69	115.46	112.13	141.35	113.7	122.89	129.58	129.36	152.15	149.01	180.25	137.38	4.2	(1.8–6.7)	0.004	—
70–74	109.43	101.15	104.7	79.34	103.95	126.65	142.52	134.65	180.79	126.06	119.36	5.3	(0.8–10.1)	0.026	—
75–79	100.5	110.42	72.9	84.34	106.38	94.41	97.85	119.69	114.48	137.6	103.66	3.6	(-0.3-7.7)	0.068	—
80–84	80.56	100.65	74.8	80.68	59.89	69.4	61.85	95.9	82.09	58.27	74.6	−2.2	(−6.8–2.5)	0.302	—
85-	69.8	116.82	31.86	36.72	41.87	46.6	60.53	81.76	40.54	62.86	56.18	−1.2	(−11.3–10.1)	0.799	—

**Table 5:** Mortality of female breast cancer in urban Shenyang in 2008–2017.

Year	Population	Deaths	Crude rate	ASR
All	18,782,956	2,710	14.43	7.43
2008	1,761,826	201	11.41	7.17
2009	1,774,839	226	12.73	8
2010	1,835,562	215	11.71	6.38
2011	1,897,625	264	13.91	7.57
2012	1,906,605	290	15.21	7.88
2013	1,901,339	269	14.15	6.95
2014	1,921,490	265	13.79	6.59
2015	1,913,126	328	17.14	8.26
2016	1,921,961	315	16.39	7.5
2017	1,948,583	337	17.29	7.99

Abbreviations: ASR, age-standardized mortality rate.

**Table 6:** APC and AAPC of crude mortality of female breast cancer in urban Shenyang in 2008–2017.

Year	APC				AAPC			Join point
	APC (95% CI)	*t* value		*p* value	AAPC (95% CI)	*t* value	*p* value	
2008–2012	5.5 (−2.7, 14.3)	1.7	0.150		4.6 (0.8, 8.5)	2.4	0.016	2012
2012–2017	3.8 (−1.9, 9.9)	1.7	0.150					

**Table 7:** AAPC of age-standardized mortality of female breast cancer in urban Shenyang in 2008–2017.

Year	AAPC			Join point
	AAPC (95% CI)	*t* value	*p* value	
2008–2012	0.8 (−1.5, 3.1)	0.8	0.457	—

**Table 8:** Age-specific mortality of female breast cancer in urban Shenyang in 2008–2017.

	2008	2009	2010	2011	2012	2013	2014	2015	2016	2017	2008–2017(overall)	AAPC	95%CI	*P* Value	Joinpoint
All	11.41	12.73	11.71	13.91	15.21	14.15	13.79	17.14	16.39	17.29	14.43				—
0–4	0	0	0	0	0	0	0	0	0	0	0	—	—	—	—
5–9	0	0	0	0	0	0	0	0	0	0	0	—	—	—	—
10–14	0	0	0	0	0	0	0	0	0	0	0	—	—	—	—
15–19	0	0	0	0	0	0	0	0	0	0	0	—	—	—	—
20–24	0	0	0	0	0.93	0	0	0	0	0	0.1	—	—	—	—
25–29	0.67	0	0.6	0.59	0	0.67	0.68	1.42	0	0.87	0.54	—	—	—	—
30–34	1.53	0.74	2.86	3.23	1.73	1.65	2.77	1.14	1.72	2.95	2.04	4.1	(−7.9–17.8)	0.468	—
35–39	2.92	2.9	5.65	5.28	4.73	3.14	0.73	4.83	3.83	3.4	3.7	−2.3	(−16.5–14.4)	0.744	—
40–44	8.61	7.98	5.36	5.24	8.79	8.17	7.19	10.56	6.79	9.4	7.8	2.4	(−3.4–8.6)	0.377	—
45–49	14.73	15.07	8.12	17.83	13.46	12.41	14.95	15.49	14.54	14.5	14.05	1.4	(−4.1–7.1)	0.589	—
50–54	27.59	31.78	22.01	22.28	20.24	17.89	13.31	18.37	19.05	23.39	21.08	−4.5	(−9.5–0.7)	0.079	—
55–59	25.23	30.75	17.59	24.01	24.32	22.62	27.76	26.51	21.59	23.16	24.16	−0.5	(−4.5–3.6)	0.782	—
60–64	21.04	21.43	22.98	22.98	34.58	24.31	21.32	34.24	38.13	28.31	28.07	5.0	(0.3–10.0)	0.040	—
65–69	26.24	28.68	28.27	41.35	30.72	25.63	26.13	32.77	27.94	34.59	30.32	0.9	(−3.0–5.0)	0.617	—
70–74	25.92	32.25	26.81	29.92	39.67	42.22	32.18	41.19	34.89	38.91	34.19	4.1	(0.6–7.7)	0.027	—
75–79	35.06	46.99	35.64	21.44	50.46	37.76	57.08	39.9	42.18	43.78	41.18	2.7	(−4.1–10.0)	0.396	—
80–84	65.46	75.49	59.21	53.78	47.41	62.46	31.99	59.94	65.3	65.33	57.57	−1.4	(−7.4–5.1)	0.634	—
85-	46.53	74.34	50.97	62.95	51.17	59.31	41.62	74.94	53.01	60	57.54	0.5	(−4.6–5.7)	0.842	—

**Table 9:** Survival rates for female breast cancer in urban Shenyang in 2008–2017.

		Age group								Total
		0–19	20–29	30–39	40–49	50–59	60–69	70–79	80-	
3 year	# Patients at risk	1	105	854	3094	4316	2323	1317	367	12377
	# Patients survived	1	91	768	2764	3768	1997	1010	197	10596
	% Survival	100	86.67	89.93	89.33	87.3	85.97	76.69	53.68	85.61

5 year	# Patients at risk	1	66	620	2309	3171	1505	958	238	8868
	# Patients survived	1	45	497	1922	2517	1164	617	100	6863
	% Survival	100	68.18	80.16	83.24	79.38	77.34	64.41	42.02	77.39

**Table 10:** Survival rates of two common breast cancer histological types in urban Shenyang from 2008 to 2013.

Survival time (Years)	Invasive ductal carcinoma (4288 cases)			Invasive lobular carcinoma (199 cases)		
	Number at risk	Rates %	95% CI	Number at risk	Rates %	95% CI
1	4097	95.55	(94.93–96.17)	195	97.99	(96.04–99.94)
2	3894	90.81	(89.95–91.67)	187	93.97	(90.66–97.28)
3	3695	86.17	(85.14–87.20)	182	91.46	(87.58–95.34)
4	3535	82.44	(81.30–83.58)	174	87.44	(82.84–92.04)
5	3405	79.41	(78.20–80.62)	170	85.43	(80.53–90.33)

**Table 11:** Cox multivariable regression analysis of survival of two common breast cancer histological types in urban Shenyang from 2008 to 2013.

Variables	*p*-value	Hazard ratio	95.0% lower CL	95.0% upper CL
Invasive ductal carcinoma versus invasive lobular carcinoma	0.045	1.461	1.009	2.115
Age	0.000	1.033	1.029	1.037

## Data Availability

The datasets used and/or analyzed during the current study are available from the corresponding author upon reasonable request.

## List of abbreviation

AAPC: Average annual percentage changes.
